# Vodou's role in Haitian mental health

**DOI:** 10.1017/gmh.2019.23

**Published:** 2019-10-18

**Authors:** E. Auguste, A. Rasmussen

**Affiliations:** Fordham University, New York, USA

**Keywords:** Haiti, intervention, mental health, Vodou

## Abstract

This paper gives an overview of Vodou's history in Haiti and how Vodou informs Haitian mental health interventions.

## Introduction

The relationship of Vodou to the mental health and identity of Haitian people is a nuanced one. While the proportion of Haitians that actually practice Vodou is hard to enumerate, most adhere to some aspects of Vodou (Brodwin, 1996; WHO/PAHO, 2010), including a substantial portion of the Haitian people that identify as Catholic or Protestant (Safran *et al*. 2011). Haitian Vodou represents a unique religious tradition that is based in African spiritualties (Nobles, 2015). Even before Haiti's inception as the first Black republic in 1804, Vodou has been an extant and powerful force in the identities of the Haitian people (Martin, 2012; Nobles, 2015). According to Sterlin (2006) while many western peoples have an anthropocentric understanding of self, in which people are in control of their own worlds, Haitian Vodou posits a cosmocentric worldview, in which people understand themselves as nested within and impacted by a larger spiritual and psychosocial context. It should be noted that many Haitians hold both Haitian Vodou and western understandings of self, and may experience some distress trying to integrate the two (Blanc & Madhère, 2017).

The distinct cultural differences between Haitian Vodou and western understandings came to the forefront of global consciousness following the 12 January 2010 earthquake in Haiti. The massive humanitarian response drew attention to an insufficient mental health structure and resulted in several foreign and Haitian-led mental health initiatives (Nicolas *et al*.y, 2012). One factor that complicated these efforts was the reliance on faith-based healers for many problems understood as psychological in North America and Europe. Vodou priests (Hougans) and priestesses (Mambos), as well as Catholic and Protestant priests are responsible for the majority of mental health care in Haiti (Méance, 2014).

In order to more fully address the mental health needs of the Haitian people, the history of Vodou in psychology and the modern importance of Vodou must be understood. Ramsey (2011, p. 1) has noted that, ‘…no religion has been subject to more… misrepresentation from outsiders over the past two centuries’. In addition, how Vodou might impact mental health services needs to be considered in order to inform more comprehensive psychologically based interventions.

## History and understanding of Haitian Vodou

The history of Haiti and a comprehension of Haitian Vodou are important in order to understand the mental health of Haitian people. In 1625, at the height of European colonialism, Spain officially ceded control of the western half of Hispaniola to France (Nobles, 2015). This territory was re-named Saint-Domingue and over the next century France increased the number of enslaved Africans in the colony from 2000 to nearly half a million (Ferguson, 1987). African people from diverse regions, including but not limited to, Nigeria, Senegal, the Congo, Benin, Ghana, and Cameroon were taken and forced to endure the barbarities of chattel slavery (Nobles, 2015). Many of these enslaved peoples came from spiritual traditions in which spirits are able to interact with and guide the living (Nobles, 2015). Vodou, an Ayizo word meaning spirit, emerged when the distinct ethnicities integrated their belief systems. As an example, the enslaved Nago people in Haiti believed their spirits knew and respected those of the Kongo people (Nobles, 2015).

Vodou features several classes of spiritual beings, the lowest of which are the *lwa*, or *peti ange*, meaning little angels, and the highest is *Le Bon Dieu*, a single creator god understood to be more removed from the daily lives of his creations (Métraux, 1958; Nobles, 2015). While Catholic figures, such as the Virgin Mary and Jesus Christ, are very present in spiritual life in Haiti, they can often be relegated to the background, while *lwa* are often at the forefront (Métraux, 1958). As such, the *lwa* are the spirits that people are able to communicate with directly. The *lwa* are divided into classes based on their origin and their influence. The classes of *lwa* are referred to as *nanchons*, or nations. There are *lwa* belonging to the Rada, believed to represent the old gods of Africa, Petro, believed to represent the malevolent spirits in Africa, Nago, believed to originate from Nigeria, Kongo, believed to originate from the Kongo, and Ghede, believed to be the spirits of the living who have passed on (Nobles, 2015; Bellegarde-Smith, 2006).

*Lwa* serve as a moral framework and represent distinct elements for the living. As an example, *Lwa Azaka* is understood to represent the family as well as the connection to the land of Haiti. As such, it is common for newly immigrated Haitian practitioners in North America and Europe to pray to him (Brown, 1991). There are *lwa* reflecting broader elements, such as the sea and love, to more traditional aspects of daily life, such as rum-making and the marketplace (Filan, 2006). It is important to note that Vodou is a constantly evolving and lively religion in which the *lwa* are capable of moving through different nations (Dayan, 1996). So while Vodou remains a structured, hierarchical religion it is just as capable of adjusting to present concerns of its practitioners.

The relationship between *lwa* and Vodou's practitioners is bidirectional. According to Denis (1956), individual identity depends on the *lwa* for protection. Specifically, every person has a *ti bon anj*, or little good angel, that is responsible for consciousness and emotions. The *ti bon anj* requires the *lwa* to keep it bonded to the individual body. The *gwo bon anj*, or big good angel, serves as a spiritual shadow for the body and is what travels in our dreams. As such, according to Dayan (1996), Vodou's conception of identity is three-part: a spiritual tether that never leaves the body (*ti bon anj*), the *lwa* as a source of protection for that tether, and a spiritual shadow that is capable of traveling through dreams (*gwo bon anj*). It is the relationship between the first two aspects of identity that enables Vodou practitioners to directly interact with the *lwa* through a *crise de possession*, or a spiritual possession. While possession has a negative connotation in the West, within Vodou possession is understood to be a divine experience in which a *lwa* is able to ‘mount’ an individual for a brief period of time. According to Mars (1966), ceremonies to bring about possessions can serve a range of purposes including maintaining positive relationships with the *lwa*, seeking guidance, and providing a treatment for various maladies.

As leaders of Vodou practice, Hougans and Mambos are responsible for learning and navigating the spiritual nations. According to Méance (2014), the main function of the Hougan and Mambo is to heal. The training period to become a Vodou healer is estimated to be 5 years. After this training period is completed, Vodou healers are entrusted to handle various illnesses reported by Vodou adherents. Hougan or Mambos will conduct a *pasé leson*, which serves as a type of diagnostic interview for help-seekers. During these interviews, practitioners are asked about their social relationships, religious piety, as well as their current and past mistakes (Méance, 2014). Once the interview is completed, the Hougan or Mambo will form a hypothesis about the origin of the practitioner's illness. Hypotheses can include *lwa* possession, back luck, or spiritual retaliation. The final step is to treat the illness with the appropriate subset of rituals for the type of illness (Méance, 2014). Treatment for ‘bad luck’ focuses on rebuilding a practitioner's confidence. According to Charles (1986) rituals for bad luck can include:

(a) cleansing the person, with ointments, oils, magical potions, bath with plants, wines and perfumes, (b) cleansing the client's environment – typically the house – with incense, candles and magical waters, and (c) construction of an amulet or special necklace that the client will have to wear for personal protection (p. 188).

In a similar vein, treatments surrounding angered *lwa* or *lwa* possession center on connecting the practitioner with the angered spirit so that he or she can atone. These ceremonies can include food or animal sacrifices as offerings to the specific *lwa*. In order to communicate with the spirit world, the Hougan or Mambo will contact *Papa Legba*. *Papa Legba* is central to all Vodou ceremonies, as he is the *lwa* responsible for overseeing the crossroads between the living and the *lwa* (Nobles, 2015).

It is important to note that there is no structured time period within which a treatment is supposed to work. Those that are in need of help are expected to remain faithful to the tradition until their problems are resolved (Méance, 2014).

## Mental illness in Haiti

Vodou provides Haitian people with a cosmocentric view of mental health (Sterlin, 2006). Within this worldview, the influence of spirits as well as one's psychosocial context directly impact his or her well-being. In Haiti, it is often said that ‘*tout maladi pa maladi dokte*’, which translates as ‘not all illnesses can be treated by doctors’. While there are still significant barriers to mental health care, it has been shown that Haitian people tend to prefer engaging with Catholic priests, Hougans, and Mambos in order to treat psychological symptoms (Méance, 2014). Several authors have tried to contextualize the experience of mental illness in Haiti by identifying local idioms of distress. Medical anthropologists have identified several general categories of disease: *maladi Bondyé* or problems of natural origin, *maladi peyi* or common medical problems, and *maladi moun fé mal* or problems with origins in magic caused by others. There are two additional categories of *maladi bon lwa* and *maladi Satan* that refer to diseases that are supernatural in origin and are sent without the intent of individuals (Kiev, 1961; Carrazana *et al*. 1999; Sterlin, 2006).

As it pertains to mental illness, psychological problems are thought to be distinguished between problems of the *tèt* (head) and the *kè* (heart) (Keys *et al*. 2012). In an ethnographic study, Keys *et al*. (2012) identified 17 idioms of distress in Haiti's Central Plateau that were categorized into emotional, cognitive, or psychosocial distress. *Tèt* and *kè* expressions made up 55% of these idioms. *Tèt* idioms were associated with forgetfulness, poor concentration, worry, and unusual behavior. *Kè* idioms were associated with emotional and physical distress and dysfunction. Other idioms like *rèflechi twop* (thinking too much) and *santi m prale* (fear) did not fit perfectly into either category. For similar work in Haiti, see Bolton *et al*. (2012).

There also exists limited research on identifying psychological disorders using Haitian idioms of distress. Rasmussen *et al*. (2014) combined emic and etic understandings of mental health symptoms in order to assess for depression in Haiti's central plateau. Participants were asked to identify common mental health symptoms using local idioms of distress. This approach allowed for a more culturally salient assessment of depressive symptoms. As highlighted by Tiberi (2016), however, the implications of possibly dismissing the perceived spiritual origin of symptoms for medical accuracy should be acknowledged and discussed. While Haitian people may not object to working with mental health workers, treatments or diagnoses that do not recognize a shared religious reality run the risk of alienating help-seeking clients.

As such, there are significant distinctions between *depression* and *depression mentale* in Haiti. The former represents a general discouragement, whereas the latter represents a construct more closely aligned with the clinical diagnosis of a major depressive disorder (WHO/PAHO, 2010). According to Hillel *et al*. (1994) *depression mentale* is characterized by an array of somatic symptoms including, but not limited to: headaches, back pain, feelings of emptiness, and fatigue. However, *depression mentale* is most often attributed to a comorbid medical condition, malnutrition, or *malady moun fé mal*. Rasmussen *et al*. (2015) also found that idioms of distress such as *santi de la la* or feeling low-energy, *santikè sere* or feeling like your heart is constricted, and *kalkile twòp* or thinking too much, were all linked to *depression mentale*. *Depression mentale* is most often treated within the family and by social support systems, and not by medical or mental health professionals (Desrosiers & Fleurose, 2002).

General psychosis must also be understood within the cosmoscentric perspective. While psychotic disorders are typically diagnosed due to hallucinatory and delusional symptoms in the West, the hallmarks of these symptoms look different in Haiti. For example, while intrusive thoughts and voices attributed to another being may be characteristic of the auditory hallucinations of schizophrenia in the U.S.A., it is unremarkable for some Haitian people to communicate with spirits throughout their daily life. According to Miller (2000), it becomes exceedingly important to consider the theme and content of symptoms to avoid incorrectly pathologizing normative religious practice as psychotic.

Due to high rates of criminal and political violence and natural disasters there is a high rate of trauma-related disorders in Haiti. According to WHO/PAHO (2010) the 2010 earthquake subjected a significant number of Haitian people to a severe trauma. In addition, it is estimated that approximately 40% of Haitian youth have experienced trauma related to physical assault, sexual assault, kidnapping, death of a family member, and gang violence (Jaimes *et al*. 2008). Haitian women are especially vulnerable to developing posttraumatic stress disorder (PTSD) as they also experience higher rates of intimate partner violence and sexual assault, as well as are vulnerable to physical assault and general community violence (Jaimes *et al*. 2008).

Blanc *et al*. (2016) analyzed the impact of Vodou on resilience and diagnoses of depression and PTSD following the 12 January 2010 earthquake. The authors assessed symptoms of depression and PTSD, as well as peritraumatic responses and resilience factors in 167 men and women. The authors found a complex relationship between Vodou faith and mental health problems. Those that understood the earthquake as divine punishment, due to the nation's history with Vodou, were more likely to suffer from severe PTSD symptoms. Vodou practitioners reported more resilience factors on average, but were more vulnerable to *depression mentale*. The authors hypothesized that this could be linked to the increase in stigma Vodou practitioners faced following the earthquake.

Beyond trauma-related disorders, the impact of historical trauma on Haitian people has been analyzed. For instance, according to Bien-Aimé (2017) the influence of Catholic worship in Vodou is evidence of a continued historical trauma. This is largely because Vodou was outlawed by the French and seen as barbaric, and Vodou practitioners had to adapt by adding aspects of Catholicism in order furtively worship. Furthermore, the continued stigma around Vodou in Haiti can be understood as internalized bigotry that is rooted in historical trauma. Specifically, some Haitian people struggle to integrate African-centered spirituality and liberation with a history of Western philosophy and religion, leading to a crisis of cultural identity (Nobles, 2015; Blanc & Madhère, 2017). While not directly linked to symptoms of trauma, the crisis of cultural identity is conceptualized to contribute to political unrest (Nobles, 2015), which in turn contributes to community violence.

In Haiti suicidality presents a schism between many health workers and the religious community. Hagaman *et al*. (2013) found that many health workers in Haiti's northern plateau did not identify suicide as a phenomenon in Haiti, despite the majority of community participants reporting it as such. The authors hypothesized that, due to its rarity in the clinical mileu, suicidal ideation was often interpreted to represent general anxiety by hospital health workers. However, as noted by many in the community, it was found that suicide was being committed regularly (Hagaman *et al*. 2013). Many Haitian people were not reporting their ideation to mental health workers, but to Vodou healers instead. In fact, those suffering from depressive and suicidal symptoms tend to prefer religious or spiritual treatment in Haiti, where the understanding of suicide is much different. That is because suicides can be completed within the context of *maladi moun fé mal*. Such a suicide can be justifiable as the deceased was not responsible for his or her actions. This, in turn, reduces the stigma of discussing suicidal ideation among Vodou practitioners.

Given the available research, it seems clear that mental illness is a concern in Haiti. Distress is verbalized in culturally unique ways and fits into a Vodou-informed cosmocentric understanding of self. When clinicians do not connect an understanding of religion in Haiti to mental health, they risk alienating and underserving those that seek treatment.

## Vodou and mental health practice

According to Casimir & Bibb (1996), some Haitian people can appear uninterested in therapy because they minimize their problems. The mental health problems that are recognized tend to be attributed to God or spirits, and so mental health professionals are not the first choice to ameliorate these problems. In addition, psychological problems and stress may not always be internalized as an individual problem. This is because spiritual forces are often understood to cause problems at the direction of envious others. In these cases people experiencing mental health problems may actually experience an increase in self-esteem as it means someone else was envious of them (WHO/PAHO, 2010). For those that experience mental health problems because of they have failed the *lwa*, their condition can be improved by fortifying their faith or through spiritual consultation (Desrosiers & Fleurose, 2002).

A survey by Wagenaar *et al*. (2012) found that three out of four rural Haitians preferred community care, which included Hougans, priests, community leaders, and herbal healers, to clinical care at hospitals. While physicians tend to believe that Haitian people are only willing to seek medical care after repeated failures from a Hougan, Khoury *et al*. (2012) found that Haitian people are open to pursing various methods of care. In one case study, a woman converted faiths twice to seek treatment for her daughter, and then brought her daughter to a medical practitioner. The researchers concluded that faith in Vodou is not nearly the obstacle that lack of access to psychiatric services represents. For example, for those that have access, psychotic disorders are typically treated with medication (WHO/PAHO, 2010). Instead, Vodou is understood to supplement available treatment and provide an explanatory model of illness.

Several mental health practitioners have attempted to understand how Vodou is able to impact mental health. The lion's share of this endeavor was undertaken and articulated by Haiti's first psychiatrist, Louis Price Mars. According to Mars (1966) Vodou constructs the ways in which mental health problems manifest, and also results in culturally specific psychological phenomena that are not disorders. Specifically, Mars (1966) noted that spiritual possession is the product of a unique ceremonial context in Vodou and cannot be understood in the absence of that. Spiritual possession, or being mounted by a *lwa*, results in observable changes in diction and posture. However, these beliefs also influence delusional thinking, such that it is common for people to suffer delusions of persecution from a nefarious individual or *lwa*. Due to the central importance of possession in Vodou, it can represent a tool for treatment. According to Charles (1986), possessions serve as context in which symptoms of delusion, depression, anxiety, and more can be addressed. Specifically, they offer a pathway towards spiritual fortification against the forces that are understood to be the sources of these problems.

Because of the role of Vodou in mental health for many Haitians there has been a call to reduce the stigma around Vodou and appreciate it as a core part of the Haitian identity (Jean-Jacques, 2012). According to Méance (2014), Western methods of psychotherapy may share several goals with Vodou. In particular, there is a mutual goal of unifying the individual with their community in a way that facilitates mental wellness. The compatibility of Vodou with psychological treatment was demonstrated in the work of Akwatu Khenti, the head of the Center for Addiction and Mental Health Office of Transformative Global Health in Canada. Khenti and his team have trained 40 Vodou priests and priestesses in cognitive behavioral therapy. This enables Hougans and Mambos to apply Vodou more specifically to patients that understand their problems as primarily rooted in the psyche (Moloney, 2015). Khenti's work shows how mental health workers might take advantage of the malleability Vodou offers in its approach a wide range of problems. In some ways parallels the religion's inception, when it first united a diverse range of faiths towards liberation.

## Conclusion

Following the 12 January 2010 earthquake in Haiti, a spotlight was placed on Haiti's complicated history with spirituality and mental health. Vodou, a religion that has integrated the ideologies and faiths of many of the ethnic groups it has come into contact with, is at the heart of this history. While it has been conventionally understood as a barrier to care among many medical professionals, it remains the primary pathway through which mental health care is addressed. Vodou is central in any understanding of mental health in Haiti. While it has been reported that inadequate access to mental health care is an extant problem in Haiti, Vodou practitioners are capable of addressing some mental health concerns. Additionally, many Haitian people believe that only Vodou can address certain problems. As psychology continues to grow as a practice in Haiti, practitioners should be optimistic that Haitian people have shown themselves to be willing to try multiple pathways to health care when there is access. Any practice of psychology should try to integrate an understanding of Vodou into its toolbox.
